# Comparative cytogenetics in three *Melipona* species (Hymenoptera: Apidae) with two divergent heterochromatic patterns

**DOI:** 10.1590/1678-4685-GMB-2017-0330

**Published:** 2018-11-29

**Authors:** Marina Souza da Cunha, Natália Martins Travenzoli, Riudo de Paiva Ferreira, Edson Kuatelela Cassinela, Henrique Barbosa da Silva, Francisco Plácido Magalhães Oliveira, Tânia Maria Fernandes Salomão, Denilce Meneses Lopes

**Affiliations:** ^1^ Laboratório de Biologia Molecular de Insetos, Departamento de Biologia Geral, Universidade Federal de Viçosa, Viçosa, MG, Brazil.; ^2^ Faculdade de Ciências Gerenciais de Manhuaçu, Campus Alfa Sul, Manhuaçu, MG, Brazil.; ^3^ Camargo Cancer Center, Centro Internacional de Ensino e Pesquisa (CIPE), São Paulo, SP, Brazil.; ^4^ Instituto de Medicina Veterinária, Universidade Federal do Pará, Campus de Castanhal, Castanhal, PA, Brazil.

**Keywords:** Chromosomal evolution, DAPI/CMA_3_ fluorochromes, Fluorescent *in situ* Hybridization (FISH), heterochromatin, Meliponini

## Abstract

The genus *Melipona* is subdivided into four subgenera based on morphological characteristics, and two groups based on cytogenetic patterns. The cytogenetic information on this genus is still scarce, therefore, the goal of this study was to characterize *Melipona paraensis, Melipona puncticollis,* and *Melipona seminigra pernigra* using the following techniques: C-banding, DAPI/CMA_3_ fluorochromes, and FISH with an 18S rDNA probe. *Melipona paraensis* (2*n*=18) and *M. seminigra pernigra* (2*n*=22) were classified as high heterochromatin content species (Group II). Their euchromatin is restricted to the ends of the chromosomes and is CMA_3_^+^; the 18S rDNA probe marked chromosome pair number 4. *Melipona puncticollis* (2*n*=18) is a low heterochromatin content species (Group I) with chromosome pair number 1 marked with CMA_3_ and 18S rDNA. Low heterochromatin content is a putative ancestral karyotype in this genus and high content is not a monophyletic trait (*Melikerria* presents species with both patterns). Differences concerning the karyotypic characteristics can be observed among *Melipona* species, revealing cytogenetic rearrangements that occurred during the evolution of this genus. Studies in other species will allow us to better understand the processes that shaped the chromatin evolution in *Melipona*.

## Introduction

Species belonging to the Meliponini tribe are also known as stingless bees. These highly eusocial bees are of pantropical distribution and are important both economically and ecologically. They produce honey and propolis, pollinate a variety of cultivated and native crops, and play an important role as providers of ecosystem services (Kerr *et al.*, 1996; Heard, 1999; Cortopassi-Laurino *et al.*, 2006; Michener, 2007). In the Neotropics, Meliponini is composed of 33 genera with approximately 417 valid species (Camargo and Pedro, 2013). Among these genera, *Melipona* Illiger 1806 is the most species-rich genus in this tribe (Silveira *et al.*, 2002), represented by 73 described species of which 43 occur in Brazil, and it is subdivided into four subgenera based on morphological characteristics: *Eomelipona, Melipona stricto sensu*, *Michmelia,* and *Melikerria* (Camargo and Pedro, 2013). It is important to highlight that a revision is needed, since *Eomelipona* is the only subgenus that was not recovered as a monophyletic clade in a molecular phylogenetic analysis (Ramírez *et al.*, 2010; Rasmussen and Cameron, 2010).

Cytogenetic studies on 22 *Melipona* species indicate a preserved autosome diploid number of *2n* = 18 chromosomes in most of the species studied so far, with *Melipona seminigra merillae* Cockerell, 1919 being the exception, showing 2*n* = 22 chromosomes (reviewed in Tavares *et al.*, 2017). Despite the conservatism in the diploid number, the *Melipona* species have a divergent pattern regarding heterochromatin content, and defined through C-banding technique it can be subdivided into two groups: Group I is comprised of species with a low content of heterochromatin, while Group II is comprised of species with a high heterochromatin content (Rocha and Pompolo, 1998; Rocha *et al.*, 2002, 2003; Lopes *et al.*, 2008, 2011). In this context, the subgenera *Eomelipona* and *Melipona stricto sensu* are comprised only of species with a low content of heterochromatin, *Michmelia* only of species with a high content, while *Melikerria* has species with both patterns. The cytogenetic data available on the genus *Melipona* regarding chromosome number, C-banding, CMA_3_, and 18S rDNA patterns is revised in Table 1.

**Table 1:** Cytogenetic data available on 22 *Melipona* species regarding their chromosome number (karyotypic formula), C-banding (high or low content), CMA_3_ and 18S rDNA patterns. Species were assigned to subgenera based on the Moure’s catalogue.

Subgenus	Species	Chromosome Number	C-Banding	CMA_3_	18S rDNA	References
*Eomelipona*	*M. asilvai*	2*n* = 18	Low content	2 interstitial markings	2 interstitial markings ^*^	Rocha and Pompolo, 1998; Rocha *et al.,* 2002; Rocha *et al.,* 2007
	*M. bicolor*	2*n* = 18	Low content	2 interstitial markings	-	Rocha and Pompolo, 1998
	*M. marginata*	2*n* = 18	Low content	2 interstitial markings	2 interstitial markings ^*^	Rocha and Pompolo, 1998; Maffei *et al.,* 2001; Rocha *et al.,* 2007
	*M. puncticollis*	2*n* = 18 (2m+14sm+2a)	Low content	2 interstitial markings	2 interstitial markings	Present study
*Melikerria*	*M. fasciculata*	2*n* = 18	High content	Terminal marks on all chromosomes	2 terminal markings^†^	Rocha *et al,* 2002; Lopes *et al.,* 2011
	*M. quinquefasciata*	2*n* = 18^‡^	Low content	2 interstitial markings	-	Rocha *et al.,* 2007
*Melipona*	*M. favosa*	2*n* = 18 (12m+4sm+2a)	-	-	-	Hoshiba, 1988
	*M. mandacaia*	2*n* = 18 (2m+14sm+2a)	Low content	2 interstitial markings	-	Rocha *et al.,* 2003
	*M. quadrifasciata*	2*n* = 18 (4m+12sm+2a)	Low content	2 interstitial markings	-	Rocha and Pompolo, 1998
	*M. subnitida*	2*n* = 18	Low content	2 interstitial markings	-	Rocha *et al.,* 2002; Rocha *et al.,* 2007
*Michmelia*	*M. capixaba*	2*n* = 18	High content	Terminal marks on all chromosomes	-	Rocha and Pompolo, 1998; Rocha *et al.,* 2002
	*M. captiosa*	2*n* = 18	High content	-	-	Rocha and Pompolo, 1998
	*M. crinita*	2*n* = 18	High content	Terminal marks on all chromosomes	-	Rocha *et al.,* 2002
	*M. flavolineata*	2*n* = 18	High content	Terminal marks on all chromosomes	-	Lopes *et al.,* 2011
	*M. fuliginosa*	2*n* = 18	High content	Terminal marks on all chromosomes	-	Lopes *et al.,* 2011
	*M. fuscopilosa*	2*n* = 18	High content	Terminal marks on all chromosomes	-	Rocha and Pompolo, 1998; Rocha *et al.,* 2002
	*M. mondury*	2*n* = 18	High content	Terminal marks on all chromosomes	-	Lopes *et al.,* 2008
	*M. paraensis*	2*n* = 18	High content	Terminal marks on all chromosomes	2 terminal markings	Present study
	*M. rufiventris*	2*n* = 18^‡^	High content	Terminal marks on all chromosomes	-	Lopes *et al.,* 2008
	*M. scutellaris*	2*n* = 18	High content	Terminal marks on all chromosomes	-	Rocha and Pompolo, 1998; Rocha *et al.,* 2002
	*M. seminigra merrillae*	2*n* = 22	Low content?^§^	-	-	Francini *et al.,* 2011
	*M. seminigra pernigra*	2*n* = 22	High content	Terminal marks on all chromosomes	2 terminal markings	Present study

^*^ Ag-NOR data.

^†^ *M. compressipes* in the paper of Rocha *et al.* (2002) is indeed *M. fasciculata* (Tavares *et al.*, 2017).

^‡^ B chromosomes were reported in these two species.

^§^ Reevaluated as high content. More details are given in the text.

The goal of this study was to describe the cytogenetic characteristics (chromosomal number, heterochromatin content, DAPI/CMA_3_ fluorochromes, and 18S rDNA patterns) of three *Melipona* species (*Melipona paraensis* Ducke, 1916, *Melipona puncticollis* Friese, 1902, and *Melipona seminigra pernigra* Friese, 1903), and to compile the cytogenetic data available for this taxon in order to identify the chromosomal variation that is characteristic for each *Melipona* Group (I and II), as well as to understand the role of these regions in the evolution of chromosomes in the genus.

## Material and Methods

The three *Melipona* species (*M. paraensis, M. puncticollis,* and *M. seminigra pernigra*) were collected in Altamira, state of Pará, Brazil. The specimens were identified by Sílvia Regina de Menezes Pedro (Universidade de São Paulo, Ribeirão Preto, Brazil), and deposited in the scientific collection of the Apiário Central at Universidade Federal de Viçosa, Viçosa, Minas Gerais, Brazil. Mitotic chromosomes were obtained from cerebral ganglia of larvae in the final defecation stage (Imai *et al.*, 1988). The conventional staining was done using Giemsa diluted in Sorensen buffer in a 1:30 proportion, and at least 25 larvae of each species were analyzed. The chromosomes were classified following the arm ratios given by Levan *et al.* (1964). Heterochromatin was visualized through C-banding (Sumner, 1972) and digital images of the metaphases were taken in a BX53F Olympus microscope equipped with a DP73F Olympus camera, using CellSens imaging software.

Sequential staining with the fluorochromes 4’-6-diamindino-2-phenylindole (DAPI) and chromomycin A_3_ (CMA_3_) was performed following the method of Schweizer (1980). Fluorescent *in situ* Hybridization (FISH) followed the protocol described by Pinkel *et al.* (1986) using a ribosomal 18S rDNA probe isolated from *M. mondury* obtained through Polymerase Chain Reaction (PCR) using the following primers: 5’-TAATTCCAGCTCCAATAG-3’ and 5’-CCACCCATAGAATCAAGA-3’. This probe was labeled by an indirect method using digoxigenin-11-dUTP (Roche Applied Science), and the signal was detected with anti-digoxigenin-rhodamine (Roche Applied Science). Digital images of the fluorescence images were captured in a BX53F Olympus microscope equipped with an MX10 Olympus camera using CellSens imaging software. An average of 10 metaphases was analyzed to determine the cytogenetic patterns revealed by the different techniques used in this study.

## Results

**Figure 1:**
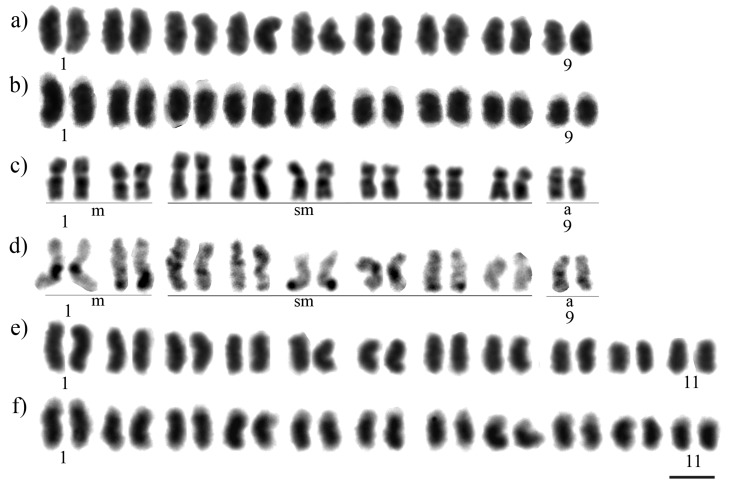
Karyotypes of *Melipona paraensis* (a - Giemsa-stained, b - C-banding); *Melipona puncticollis* (c - Giemsa-stained, d - C-banding); and *Melipona seminigra pernigra* (e - Giemsa-stained, f - C-banding). Scale bar = 5 μm.

**Figure 2:**
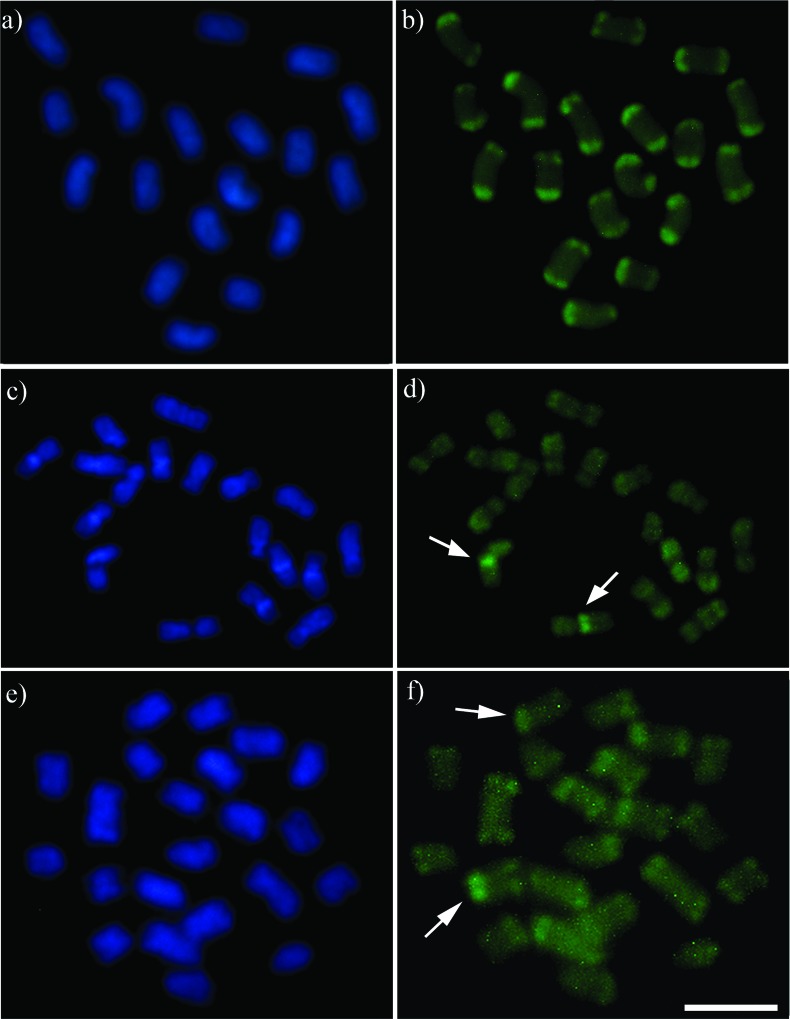
Sequential staining with DAPI/CMA_3_ fluorochromes: *Melipona paraensis* (a - DAPI, b - CMA_3_); *Melipona puncticollis* (c - DAPI, d - CMA_3_); and *Melipona seminigra pernigra* (e - DAPI, f - CMA_3_). The arrows indicate the organizing region of the nucleoli. Scale bar = 5 μm.

**Figure 3:**
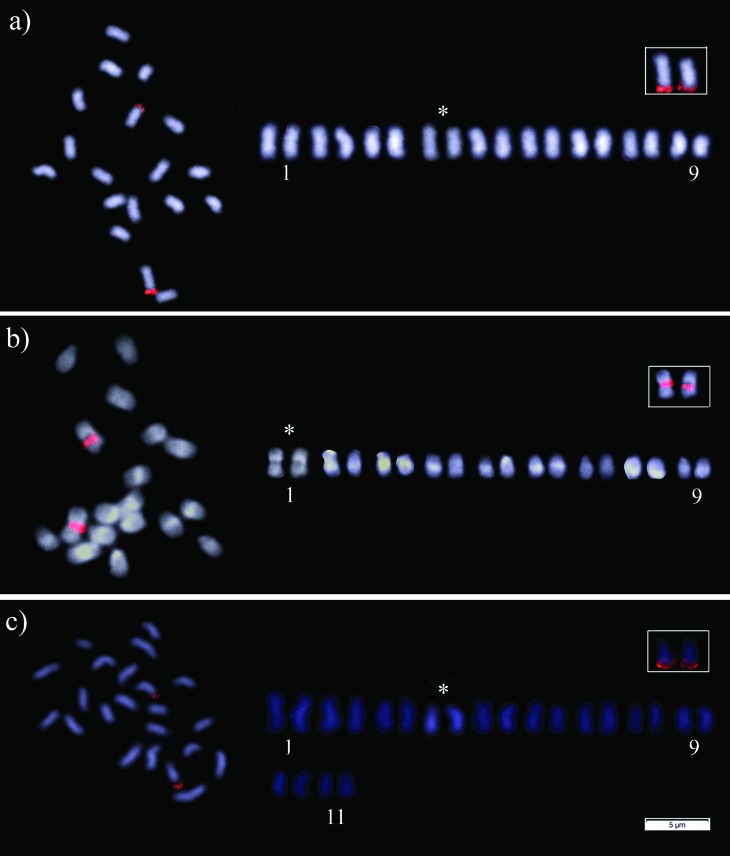
Fluorescent *in situ* hybridization (FISH) pattern with 18S rDNA probe: metaphase cells and arranged karyotype of (a) *Melipona paraensis*; (b) *Melipona puncticollis*; and (c) *Melipona seminigra pernigra*. * denotes chromosome pair marked by the probe indicated in the box. Scale bar = 5 μm.

The diploid chromosome number of *M. paraensis* was defined as 2*n* = 18 (Figure 1a). C-banding revealed that the major part of each chromosome is comprised of heterochromatin. This hindered the visualization of centromeres, and hence it was not possible to define the karyotypic formula (Figure 1b). The DAPI/CMA_3_ analysis indicated that the heterochromatin is DAPI^+^ (Figure 2a and Figure S1a-c), while CMA_3_^+^ marked all the extremities of the chromosomes corresponding to the euchromatin region (Figure 2b). FISH with 18S rDNA probe marked chromosome pair number 4 in its terminal position (Figure 3a).

The diploid chromosome number of *M. puncticollis* was defined as 2*n* = 18 and its karyotypic formula as 2*n* = 2m + 14sm + 2a (Figure 1c). C-banding indicated a low content of heterochromatin that is restricted to the pericentromeric region of chromosome pair numbers 1, 3, 6, 9, and the subtelomeric region of the long arms of chromosome pair numbers 2, 4, 5 and 7, while chromosome pair number 8 is completely euchromatic (Figure 1d). Sequential staining with DAPI/CMA_3_ fluorochromes indicated strong DAPI^+^ bands corresponding to the heterochromatin region (Figure 2c), while CMA_3_ marked the interstitial region of chromosome pair number 1 (Figure 2d). The same result was found with the 18S rDNA FISH probe (Figure 3b).

The diploid chromosome number of *M. seminigra pernigra* was defined as 2*n* = 22 (Figure 1e). C-banding revealed that the majority of each chromosome is composed of heterochromatin, hindering the visualization of the centromeres, so it was not possible to define the karyotypic formula (Figure 1f). DAPI/CMA_3_ analysis indicated that the heterochromatin is DAPI^+^ (Figure 2e and Figure S1d-f). CMA_3_^+^ marked all the extremities of the chromosomes corresponding to the euchromatin region, and we could identify one chromosome pair that strongly stained with CMA_3_ fluorochrome (Figure 2f). FISH with the 18S rDNA probe marked chromosome pair number 4 in its terminal position (Figure 3c).

## Discussion

The three species analyzed in this study presented distinct chromosome numbers: *M.* (*Michmelia*) *paraensis* and *M.* (*Eomelipona*) *puncticollis* presented 2*n* = 18 chromosomes, while *M. (Michmelia) seminigra pernigra* showed 2*n* = 22 chromosomes. The autosomal chromosome number that prevails in the genus *Melipona* is 2*n* = 18, but a few exceptions can be found, such as in *M. (Michmelia) seminigra merillae* that also has 2*n* = 22, and *Melipona (Melikerria) quinquefasciata* Lepeletier, 1836 and *Melipona (Michmelia) rufiventris* Lepeletier, 1836, both of which have 2*n* = 18 autosomal chromosomes, but they present variation with respect to the number of B chromosomes found in different populations (reviewed in Tavares *et al.*, 2017).

Regardless of the conservatism in the diploid number, differences concerning the karyotypic formula and heterochromatin content could be observed among species, revealing cytogenetic rearrangements that have occurred during the evolution of the genus. Changes in the karyotypic formula among species belonging to Group I indicate the occurrence of pericentric inversions that altered the number of metacentric and submetacentric chromosomes in this group: *Melipona favosa* Fabricius, 1798 (2*n* = 12m + 4sm + 2a) (Hoshiba, 1988), *Melipona mandacaia* Smith, 1863 (2*n* = 2m + 14sm + 2a) (Rocha *et al.*, 2003), *M. puncticollis* (2*n* = 2m + 14sm + 2a) (present study), and *Melipona quadrifasciata* Lepeletier, 1836 (2*n* = 4m + 12sm + 2a) (Silva *et al.*, 2012). In Group II species, the high heterochromatin content masks the position of the centromere, and therefore, makes it difficult to identify the morphology of the chromosomes to define the karyotypic formula of these species. This is a common trait among the *Melipona* species belonging to Group II, rather than an issue related to the quality of the metaphases (Rocha *et al.*, 2002; Lopes *et al.*, 2008, 2011).

In the species analyzed in this study, *M. puncticollis* is a low heterochromatin content species, while *M. paraensis* and *M. seminigra pernigra* are high heterochromatin content species (Figure 1). The first description of the C-banding pattern on *M. seminigra merrilae* indicated this subspecies as part of Group I, with low heterochromatin content (Francini *et al.*, 2011), but analyzing the images from that publication, the pattern seems to be very similar to the high heterochromatin content species, as it was not possible to visualize the centromeres, and they had heterochromatin as the predominant constituent of the chromosomes. Ongoing cytogenetic studies on this subspecies confirm that *M. seminigra merrillae,* as well as *M. seminigra pernigra* are high heterochromatin content subspecies belonging to Group II (unpublished data).

In eusocial bees, the heterochromatin is usually AT-rich (DAPI^+^) (Brito *et al.*, 2003; Rocha *et al.*, 2003; Lopes *et al.*, 2011; Godoy *et al.*, 2013), and this is a pattern shared by *Melipona* species with both low and high heterochromatin content (Figure 2). CMA_3_^+^ positive bands are another characteristic used to distinguish Groups I and II in this genus (Table 1): Group I species have only one chromosome pair CMA_3_^+^ marked in its interstitial position, and this chromosome pair is usually related to the nucleolar organizing region (NOR) (Rocha *et al.*, 2002), indicating that the NOR is CG-rich in this group; Group II species have CMA_3_^+^ terminal markings on all of the chromosomes corresponding to the euchromatin, indicating that these regions are CG-rich, and in some cases it is possible to identify one pair with the brightest mark as associated with ribosomal cistrons, as for instance in *M. seminigra pernigra* (Figure 2F) and other high heterochromatic content species (Lopes *et al.*, 2008, 2011). It is interesting to note that the solitary bee *Melitoma segmentaria* Fabricius, 1804 has the opposite pattern, as the euchromatic portion of the chromosomes are CMA_3_^-^ and the heterochromatic ones are CMA_3_^+^ (Cristiano *et al.*, 2014). Other solitary bees, such as *Euglossa townsendi* Cockerell, 1904 and *Euglossa carolina* Linnaeus, 1758), have the same cytogenetic divergence as *Melipona* with regard to the heterochromatin content (species with low and high heterochromatin content), but they have unique CMA_3_ accumulation patterns, showing that the heterochromatin is heterogeneous with respect to its composition, with some blocks rich in AT and others rich in CG (Fernandes *et al.*, 2013), highlighting the diversity of the patterns observed among bees.

To this date, there is only one report that has used FISH to confirm the position of the NORs with an 18S rDNA FISH probe; this was done in *Melipona fasciculata* Smith, 1854 (Rocha *et al.*, 2002, revised in Tavares *et al.*, 2017). Together with our study, it seems that only one pair of chromosomes labeled with this probe, which can be considered as a conserved characteristic in this genus (Figure 3). Studies applying ribosomal probes in bees are still scarce, but analyses combining Ag-NOR, CMA_3_^+^ bands, and FISH 18S rDNA techniques have been used to identify NORs in different Meliponini species (Rocha *et al.*, 2002; Brito *et al.*, 2005; Duarte *et al.*, 2009; Krinski *et al.*, 2010; Lopes *et al.*, 2011; Godoy *et al.*, 2013; Miranda *et al.*, 2013). Based on these three different techniques it can be inferred that having only one pair of NORs is a conserved characteristic in *Melipona* (Table 1).

Regardless of the conservatism in the number of markings, the position of the 18S rDNA cistrons can be used to differentiate Groups I and II in *Melipona*, as they are interstitial in low content species and terminal in high content ones (Table 1). Independent of the technique applied, the literature indicates pair number 1 as the NOR bearer in this genus. For: *Melipona asilvai* Moure, 1971, *M. mandacaia,* and *Melipona marginata* Moure, 1992 this was inferred by Ag-NOR (Maffei *et al.*, 2001; Rocha *et al.*, 2002, 2003). For *Melipona bicolor* Lepeletier, 1836, *Melipona capixaba* Moure and Camargo, 1994, *Melipona mondury* Smith, 1863, *M. quadrifasciata*, *M. quinquefasciata, M. rufiventris,* and *Melipona subnitida* Ducke, 1910 the identification was done by CMA_3_ fluorochrome (Rocha *et al.*, 2007; Lopes *et al.*, 2008), and for *M. fasciculata* it was identified by FISH with a ribosomal probe (Rocha *et al.*, 2002 revised in Tavares *et al.*, 2017). However, in our study only the low content species *M. puncticollis* had the first pair as the NOR bearer, while the high content species *M. paraensis* and *M. seminigra pernigra* had pair number 4 marked with the 18S rDNA probe (Figure 3), highlighting another distinct characteristic between Groups I and II. We argue that none of the cited studies above arranged the karyotype. Hence in metaphase cells, the terminal location of the probe in the high content species might have given the impression of a bigger chromosome (see Figure 3).

Despite being polyphyletic, basal *Eomelipona* species group together with *Melipona stricto sensu* (Ramírez *et al.*, 2010), and this clade is composed of species with low heterochromatin content (Table 1), indicating that this is the plesiomorphic characteristic of the genus, while high heterochromatin content appeared more than once during the evolution and diversification of this taxon, emerging in both *Melikerria* and *Michmelia* subgenera. As we could observe both heterochromatin patterns in *Melikerria* (Table 1), the classification of the *Melipona* species into low and high heterochromatin content species did not form natural groups and did not represent monophyletic clades in the phylogenetic analysis.

The current study aimed to describe three *Melipona* species with divergent patterns of heterochromatin accumulation, arguing that a karyotype with low heterochromatin content is a putative ancestral karyotype in this genus and that high heterochromatin content is not a monophyletic characteristic. We also contributed with new cytogenetic data on Groups I and II, highlighting the cytogenetic rearrangements that occurred during the chromosome evolution in this major stingless bee genus. Finally, we emphasize the importance of cytogenetic analyses to evidence the chromosomal rearrangements that occurred during the evolution of different species (Imai *et al.*, 1994; Menezes *et al.*, 2014). Studies in other species will allow us to better understand the processes that shaped chromatin evolution in *Melipona*.

## Supplementary material

The following online material is available for this article:

Figure S1 - DAPI stained metaphases of *Melipona paraensis Melipona seminigra pernigra*.

## References

[B1] Brito RM, Caixeiro APA, Pompolo SG, Azevedo GG (2003). Cytogenetic data of *Partamona peckolti* (Hymenoptera, Apidae, Meliponini) by C banding and fluorochrome staining with DA/ CMA_3_ and DA/DAPI. Genet Mol Biol.

[B2] Brito RM, Pompolo SG, Magalhães MFM, Barros EG, Sakamoto-Hojo ET (2005). Cytogenetic characterization of two *Partamona* species (Hymenoptera, Apidae, Meliponini) by fluorochrome staining and localization of 18 S rDNA clusters by FISH. Cytologia.

[B3] Camargo JMF, Pedro SRM, Moure JS, Urban D, Melo GAR (2013). Meliponini Lepeletier, 1836. Catalogue of Bees (Hymenoptera, Apoidea) in the Neotropical Region.

[B4] Cortopassi-Laurino M, Imperatriz-Fonseca VL, Roubik DW, Dolin A, Heard T, Aguilar I, Venturieri GC, Eardley C, Nogueira-Neto P (2006). Global meliponiculture: Challenges and opportunities. Apidologie.

[B5] Cristiano MP, Simoes TG, Lopes DM, Pompolo SG (2014). Cytogenetics of *Melitoma segmentaria* (Fabricius, 1804) (Hymenoptera, Apidae) reveals differences in the characteristics of heterochromatin in bees. Comp Cytogenet.

[B6] Duarte OMP, Martins CCC, Waldschmidt AM (2009). Occurrence of multiple nucleolus organizer regions and intraspecific karyotype variation in *Scaptotrigona xanthotricha* Moure (Hymenoptera, Meliponini). Genet Mol Res.

[B7] Fernandes A, Werneck HA, Pompolo SG, Lopes DM (2013). Evidence of separate karyotype evolutionary pathway in *Euglossa* orchid bees by cytogenetic analyses. An Acad Bras Cienc.

[B8] Francini IB, Gross MC, Nunes-Silva CG, Carvalho-Zilse GA (2011). Cytogenetic analysis of the Amazon stingless bee *Melipona seminigra merrillae* reveals different chromosome number for the genus. Sci Agr.

[B9] Godoy DC, Ferreira RP, Lopes DM (2013). Chromosomal variation and cytogenetics of *Plebeia lucii* and *P. phrynostoma* (Hymenoptera: Apidae). Fla Entomol.

[B10] Heard TA (1999). The role of stingless bees in crop pollination. Annu Rev Entomol.

[B11] Hoshiba H (1988). Karyological analysis of a stingless bee, *Melipona favosa* (Apidae, Hymenoptera). Cytologia.

[B12] Imai HT, Taylor RW, Crosland MW, Crozier RH (1988). Modes of spontaneous chromosomal mutation and karyotype evolution in ants with reference to the minimum interaction hypothesis. Jpn J Genet.

[B13] Imai HT, Taylor RW, Crozier RH (1994). Experimental bases for the minimum interaction theory. I. Chromosome evolution in ants of the *Myrmecia pilosula* species complex (Hymenoptera: Formicidae: Myrmeciinae). Jpn J Genet.

[B14] Kerr WE, Carvalho GA, Nascimento VA (1996). Abelha Uruçu - Biologia, Manejo e Conservação.

[B15] Krinski D, Fernandes A, Rocha MP, Pompolo SG (2010). Karyotypic description of the stingless bee *Oxytrigona* cf. *flaveola* (Hymenoptera, Apidae, Meliponina) of a colony from Tangará da Serra, Mato Grosso State, Brazil. Genet Mol Biol.

[B16] Levan A, Fredga K, Sandberg AA (1964). Nomenclature for centromeric position on chromosomes. Hereditas.

[B17] Lopes DM, Pompolo SG, Campos LAO, Tavares MG (2008). Cytogenetic characterization of *Melipona rufiventris* Lepeletier 1836 and *Melipona mondury* Smith 1863 (Hymenoptera, Apidae) by C banding and fluorochromes staining. Genet Mol Biol.

[B18] Lopes DM, Fernandes A, Praça-Fontes MM, Werneck HDA, Resende HC, Campos LAO (2011). Cytogenetics of three *Melipona* species (Hymenoptera, Apidae, Meliponini). Sociobiology.

[B19] Maffei EMD, Pompolo SG, Silva-Junior JC, Caixeiro APA, Rocha MP, Dergam JA (2001). Silver staining of nucleolar organizer regions (NORs) in some species of Hymenoptera (bees and parasitic wasps) and Coleoptera (lady beetle). Cytobios.

[B20] Menezes RST, Carvalho JPSO, Silva TS, Somovilla A, Costa MA (2014). Evolutionary trends in the chromosome numbers of swarm-founding social wasps. Insectes Soc.

[B21] Michener CD (2007). The bees of the world.

[B22] Miranda RV, Fernandes A, Lopes DM (2013). Karyotype description of *Cephalotrigona femorata* Smith (Hymenoptera: Apidae) and the C-banding pattern as a specific marker for *Cephalotrigona*. Sociobiology.

[B23] Pinkel D, Straume T, Gray JW (1986). Cytogenetic analysis using quantitative, high sensitivity, fluorescence hybridization. Proc Natl Acad Sci U S A.

[B24] Ramírez SR, Nieh JC, Quental TB, Roubik DW, Imperatriz-Fonseca VL, Pierce NE (2010). A molecular phylogeny of the stingless bee genus *Melipona* (Hymenoptera: Apidae). Mol Phylogenet Evol.

[B25] Rasmussen C, Cameron SA (2010). Global stingless bee phylogeny supports ancient divergence, vicariance, and long distance dispersal. Biol J Linn Soc.

[B26] Rocha MP, Pompolo SG (1998). Karyotypes and heterochromatin variation (C-bands) in *Melipona* species (Hymenoptera, Apidae, Meliponinae). Genet Mol Biol.

[B27] Rocha MP, Pompolo SG, Dergam JA, Fernandes A, Campos LAO (2002). DNA characterization and karyotypic evolution in the bee genus *Melipona* (Hymenoptera, Meliponini). Hereditas.

[B28] Rocha MP, Cruz MP, Fernandes A, Waldschmidt AM, Silva-Junior JC, Pompolo SG (2003). Longitudinal differentiation in *Melipona mandacaia* (Hymenoptera, Meliponini) chromosomes. Hereditas.

[B29] Rocha MP, Pompolo SG, Fernandes A, Campos LAO (2007). *Melipona*: Six decade of cytogenetic. Biosci J.

[B30] Schweizer D (1980). Simultaneous fluorescent staining of R bands and specific heterochromatic regions (DA – DAPI bands) in human chromosomes. Cytogenet Cell Genet.

[B31] Silva WRT, Araújo ED, Scher R (2012). Caracterização do cariótipo de uma população de abelhas *Melipona quadrifasciata* (Hymenoptera: Meliponini), no município de Brejo Grande/SE. Scientia Plena.

[B32] Silveira FA, Melo GAR, Almeida EAB (2002). Abelhas Brasileiras: Sistemática e Identificação.

[B33] Sumner AT (1972). A simple technique for demonstrating centromeric heterochromatin. Exp Cell Res.

[B34] Tavares MG, Lopes DM, Campos LAO (2017). An overview of cytogenetics of the tribe Meliponini (Hymenoptera: Apidae). Genetica.

