# Supplementary material for: Comparative cytogenetics in three Melipona species (Hymenoptera: Apidae) with two divergent heterochromatic patterns
Source: Genet Mol Biol. 2018 Nov 29;41(4):806–13. doi: 10.1590/1678-4685-GMB-2017-0330 (PMC6415597; doi:10.1590/1678-4685-GMB-2017-0330)
Supplement: Supplementary file 1 [file 1415-4757-GMB-1678-4685-GMB-2017-0330-s001.pdf]

Supplementary Material to "Comparative cytogenetics in three Melipona species (Hymenoptera: Apidae) with two divergent heterochromatic patterns"

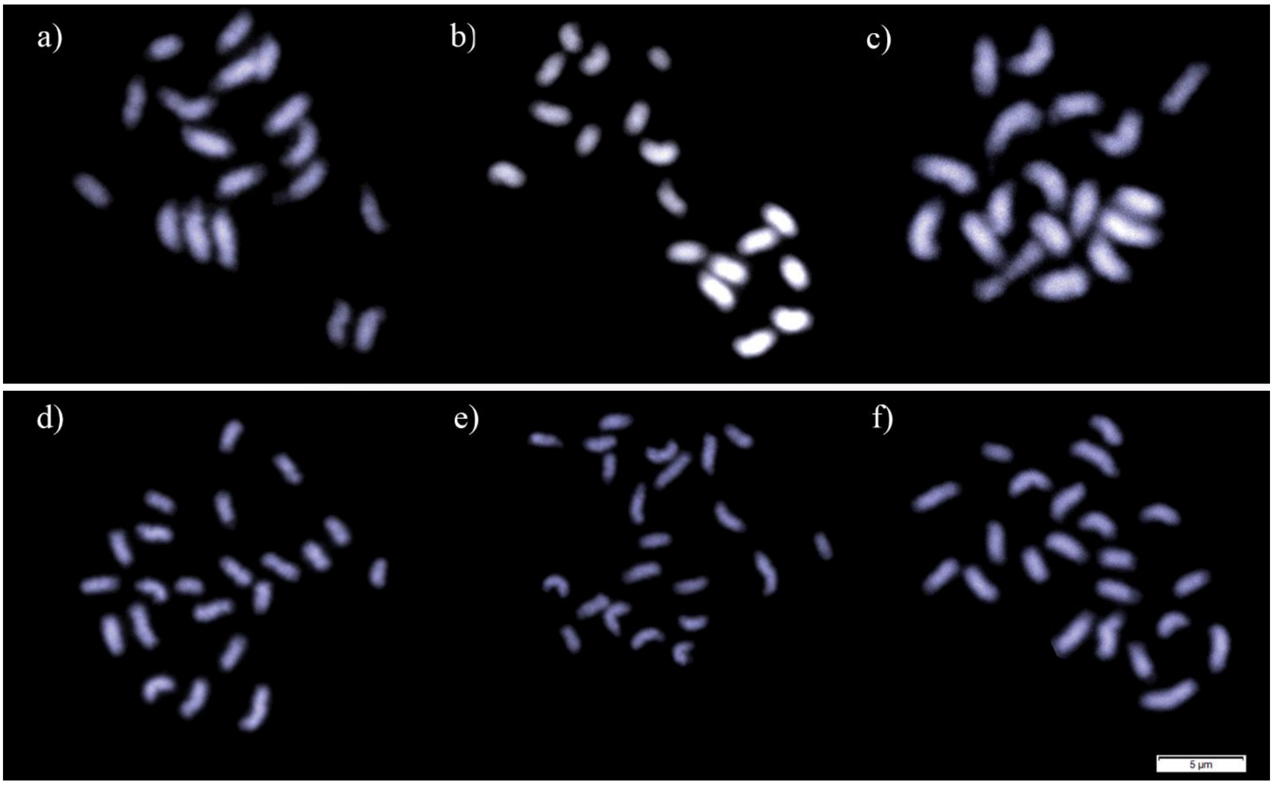

Figure S1 – DAPI stained metaphases of *Melipona paraensis* (a-c) and *Melipona seminigra pernigra* (d-f). Scale bar = 5 µm.
